# Spinal Cord Compression Due to Tophaceous Vertebral Gout: A Case Report

**DOI:** 10.7759/cureus.27101

**Published:** 2022-07-21

**Authors:** Duniel Abreu Casas, Orestes R López-Piloto, Norbery J Rodríguez de la Paz, José M Plasencia-Leonardo, Daniel Íñiguez-Avendaño, Joel V Gutierrez

**Affiliations:** 1 Department of Neurosurgery, Cuban Institute of Neurology and Neurosurgery, Havana, CUB; 2 Department of Neurosurgery and Neurophysiology, Cuban Institute of Neurology and Neurosurgery, Havana, CUB

**Keywords:** gouty tophus, gout, spinal cord compression, paraparesis, radicular pain

## Abstract

Gout tophi are deposits of urate crystals in subcutaneous tissues and joints which commonly affect the small joints of the feet and hands, causing painful arthritis. The axial skeleton is considered to be seldom affected by gout arthritis. Here we describe the clinical, MRI, and pathologic findings of a 61-year-old male patient with a previous diagnosis of gout who presented with progressive paraparesis and radicular pain. MRI showed extradural masses compressing the spinal cord and roots at two spinal levels. Two surgical interventions were performed to remove these extradural masses, which were pathologically identified as gout tophi. Pain and paraparesis had clinical improvement after surgery. This report highlights that gout can be a cause of paraparesis.

## Introduction

Gout is a metabolic disorder caused by the accumulation of uric acid in extracellular fluids [1,2]. Gout is estimated to affect one or two persons in every 1000 inhabitants [2]. Gout tophi are caused by the accumulation of monosodium urate crystals, surrounded by inflammatory cells and connective tissue, in several regions of the body [2]. Small joints and subcutaneous tissues are frequently affected, causing chronic joint pain [2]. On the other hand, the presence of gout tophi in axial joints is considered very rare and can produce neurological disorders [3]. In the vertebral column, the gout tophi can affect the joint facets, the vertebral bodies, the pedicles, and the flavum ligament [2-4]. There are just a few case reports about spinal cord disorders caused by the accumulation of monosodium urate crystals in the vertebral column joints. However, axial gout is likely more frequent than usually perceived [3,4]. Here we describe the clinical, MRI, and pathologic findings of a patient with gout and paraparesis caused by this unusual presentation of gout tophi.

## Case presentation

A 61-year-old male patient with a history of suffering from gout arthritis for 15 years attended the outpatient clinic of neurosurgery complaining of back pain and decreased muscle strength in both lower limbs for several months. The patient also had diabetes mellitus, arterial hypertension, and heart failure. He recognized that he was not following the diet recommendations or taking the medication (allopurinol, 200 mg per day) prescribed by his doctors to treat hyperuricemia. As a result, he had suffered from low back pain with paresthesia for five months and decreased muscle strength with spasticity in the lower extremities for two months, first in the left and later in the right limb. All these manifestations progressively worsened and eventually made him unable to walk and perform daily life activities.

The general physical exam revealed the presence of gout tophi in the joints of the elbows and hands, the right auricular lobe, and the right knee. His BMI was 34.3, which was consistent with grade I obesity. The neurological physical exam showed fasciculations, brisk knee and Achilles tendon reflexes, clonus, decreased muscle power (3/5), and spasticity in both lower limbs. No sphincter disorders were reported. Table 1 shows the results of the laboratory tests performed upon admission to the hospital.

**Table 1:** Results of laboratory tests.

Laboratory tests	Results	Normal range	Interpretation
Hemoglobin	13.1 g/d	12-16 g/L	Normal
Platelet count	386 x 10⁹	150-400 x 10⁹	Normal
Bleeding time	1 minute	1-4 minutes	Normal
Clotting time	7 minutes	5-10 minutes	Normal
International normalised ratio (INR)	0.98	<1.1	Normal
Glycemia	15.9 mmol/L	4.2-6.11 mmol/L	Increased
Creatinine	98.9 µmol/L	47-113 µmol/L	Normal
Uric acid	471 mmol/L	208-428 umol/L	Increased
Triglycerides	6.62 mmol/L	0.68-1.88 mmol/L	Increased
Cholesterol	9.53 mmol/L	2.9-5.2 mmol/L	Increased

**Figure 1:**
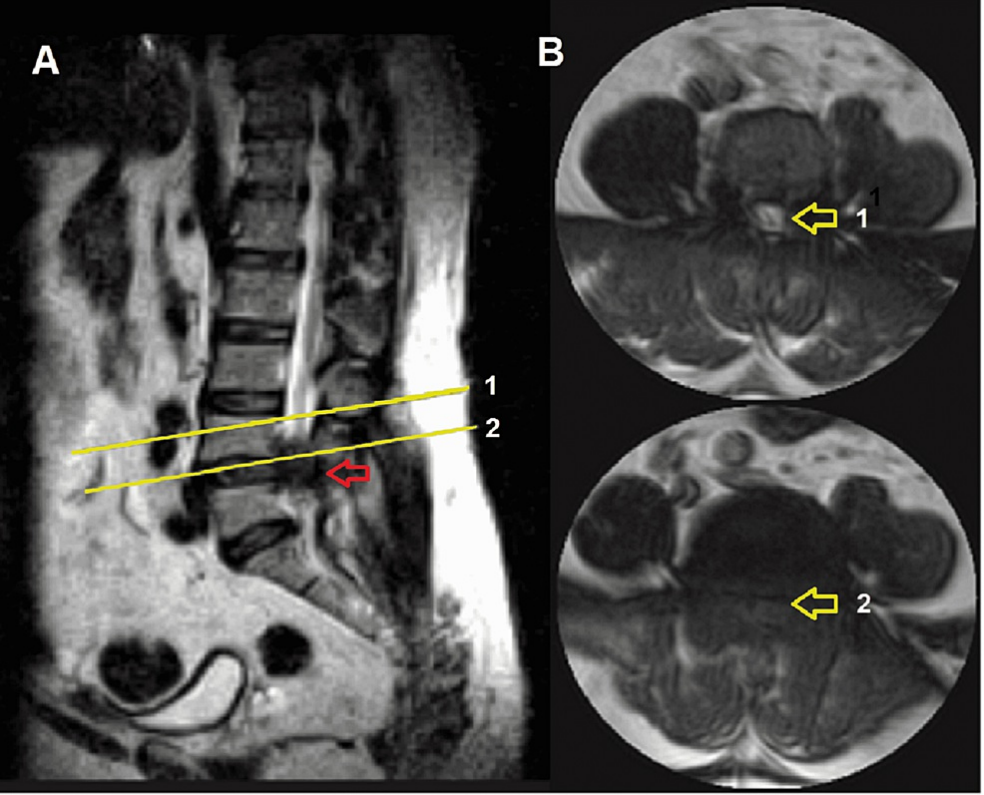
MRI of the lumbar spine. Sagittal (A) and axial (B) views in T2 sequence. The red arrow shows the tophus as a hypointense mass obliterating the spinal canal. The yellow arrows indicate: 1: axial section above the level of the lesion showing a normal spinal canal. 2: axial section at the level of the lesion showing complete obliteration of the spinal canal.

Axial and sagittal MRI views showed facet hypertrophy at right T7-T8, left T9-T10, and bilateral T11-T12 levels. At the level of T11-T12, the facet hypertrophy protruded into the spinal canal, compressing the spinal cord. Signs of spinal cord ischemia and narrowing of the posterior cerebrospinal fluid column were observed at this level. Facet hypertrophy was also identified at the L4-L5 vertebral level, associated with a hypodense lesion that occupied the spinal canal, narrowing the lateral recesses of the spinal cord (Figure 1).

Considering the patient’s background, it was hypothesized that these lesions could be related to underlying gout and explain the neurological symptoms found in the lower limbs. Therefore a surgical intervention was proposed.

Once admitted to the hospital, the patient was treated with allopurinol, one tablet (100 mg) every 8 hours. The surgical approach was planned in two steps: the first to remove the dorsal lesion at T10-T11 and the second to remove the lumbar lesion at L4-L5.

**Figure 2:**
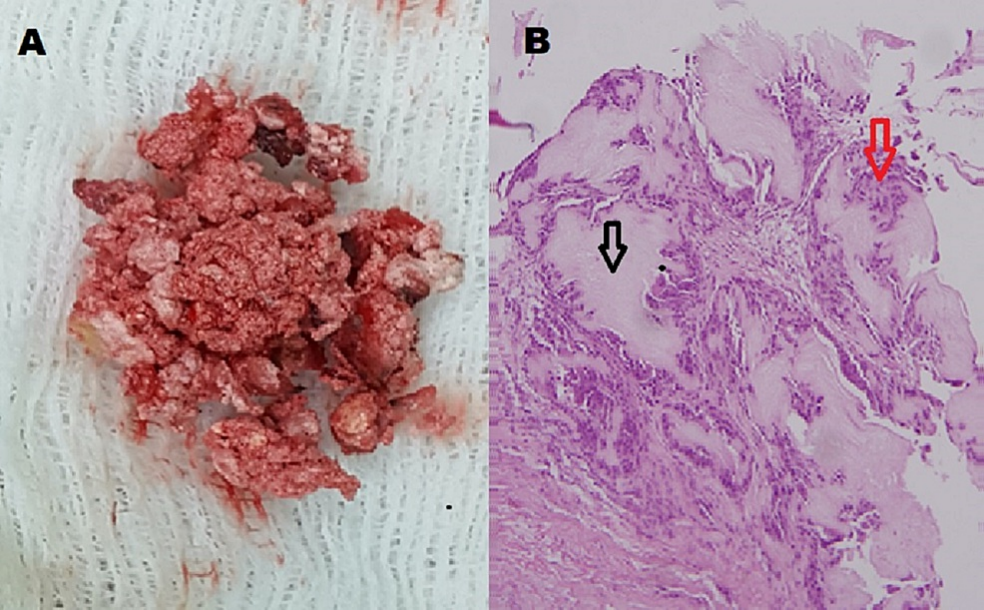
Pathologic analysis of the lesions removed from the spinal canal. The macroscopic exam (A) displayed brownish white lesions with a jelly consistency. The microscopic analysis (B) showed fragments of fibromuscular tissue, urate crystals (black arrow), and surrounding inflammatory infiltrates (red arrow).

A decompressive laminectomy with exeresis of the lesion was performed in the first surgical step. Multiple brownish white lesions with a jelly consistency were removed from within the spinal canal. The microscopic analysis demonstrated that these lesions were composed of gouty fibrocartilaginous tophi with calcifications and crystals surrounded by inflammatory infiltrates (Figure 2) invading the bone laminae and fibrocartilage of vertebrae.

After surgery, the pain decreased, but paraparesis continued with muscle strength maintained at 3/5 in both lower limbs. The patient was discharged 13 days after surgery under pharmacological treatment with colchicine (1 mg per day), allopurinol (300 mg per day), and a special diet. For the following months, the patient showed no further improvement in his neurological manifestations.

The second surgery was completed five months later to remove the lumbar lesions. A laminectomy was performed at L4 and L5 levels. A medial facetectomy and foraminotomy were performed, and dural theca was left free. A white lesion with a semi-hard appearance was removed at this level. The macroscopic exam of the bone removed showed several grayish fibroelastic fragments with whitish stippling. The microscopic study evidenced fragments of fibromuscular tissue with inflammation and fibrocartilage tissue with crystals, surrounded by an inflammatory infiltrate, suggesting gout tophus.

The patient was discharged with a follow-up in the outpatient clinic. Unfortunately, despite receiving intensive physical therapy, the muscle strength recovered only partially. Currently, the patient remains under strict control of his hyperuricemia and continues with rehabilitation therapy.

## Discussion

The present report summarizes the clinical findings and the ancillary test results of a patient affected by gout tophi in the spinal canal. Gout is a relatively common disease that can appear in five clinical forms: asymptomatic, hyperuricemic, gouty arthritis, intercritical gout, and chronic tophaceous gout [4,5]. In the chronic tophaceous gout variant, hyperuricemic levels are maintained over a long period of time, facilitating the appearance of gout tophi in different joints [1,2]. Tophaceous gout is associated with severe damage to joint cartilages, bones, and tendons, negatively impacting several aspects of the patient’s life [1,2].

The uric acid deposits are commonly found in the metatarsophalangeal joints, ankles, knees, wrists, fingers, and pinna [6]. The involvement of the axial skeleton is considered very rare, but it could be more frequent than usually recognized [2,3]. In addition, a small proportion of gout tophi can appear at the intervertebral joints and affect the spinal cord producing neurological deficits. Our patient had suffered hyperuricemia and gout arthritis for 15 years which, in association with his neurological symptoms, suggested evaluating the presence of intraspinal tophi.

In 1950, Kersley GD et al. reported the first case of spinal cord compression due to a subluxation of the first cervical vertebra in a young patient with severe gout [7]. The necropsy of that patient showed that the tophi had eroded the first cervical vertebrae causing a pathological fracture of the atlas with protrusion of the odontoid process into the foramen magnum. Since Kersley’s original article, a few case reports of patients with spinal cord involvement caused by gout tophi have been published [5,6,8-16].

In the vertebral column, gout tophi can affect different anatomical components, such as the articular facets, the vertebral bodies, the pedicles, the laminae, and the yellow ligaments. The lateral parts of the vertebra are usually more affected than the central areas [13-15]. The patient here described showed facet hypertrophy at the dorsal and lumbar levels with signs of spinal cord compression.

Spinal gouty tophi are frequently associated with neurological symptoms like pain, paralysis of the lower limbs, and urinary retention. General symptoms like fever, leukocytosis, and elevated systemic inflammatory markers are also commonly seen in these patients [14,15]. It is interesting, however, that gout duration did not correlate with axial gout [12]. The patient described in the present report had signs of corticospinal compression in both lower limbs, which were caused by the compression of the dorsal spinal cord.

Koskoff YD et al. described the first case of extradural gout at the thoracic level, causing paraplegia [8]. The radiographic prevalence of gout in the vertebral column has been estimated to be between 14% and 35% in patients with poor hyperuricemic control [9], but these estimates may be inaccurate. Intraspinal tophi can affect any segment of the spine. Toprover M et al., in their review of cases of axial gout tophi, described that 44% of the axial tophi involved the lumbar spine, 39% involved the cervical region, and 17% involved the thoracic vertebral bodies. In addition, more than 80% of the patients with axial gout have lesions at multiple spine levels [10-12]. Congruent with these data, our patient showed lesions at several spine levels.

The imaging diagnosis of intraspinal tophi is often difficult. Radiographic imaging and MRI are frequently used as the initial evaluation; however, these studies are less specific than CT in assessing intraspinal tophi. Most of the abnormalities identified on plain radiographs are nonspecific for intraspinal gout [10-12]. CT scan is more useful to evaluate the spine than plain X-rays and can be used to evaluate tophi density, which presents an attenuation of 160-170 HU [6]. Dual-energy CT (DECT) is more sensitive and specific for identifying monosodium urate deposits, but this method is not as widely available as conventional CT [6,10,17]. In our case, the lesions were identified only by axial and sagittal MRI evaluations.

Intraspinal gout tophi can be misdiagnosed as spondylitis, epidural abscess, neoplastic lesion, or degenerative stenosis [16-19]. The presence of clinical signs of spinal cord involvement in a patient with increased uric acid should suggest looking for intraspinal tophi; however, the definitive diagnosis for intraspinal tophi requires a histopathological examination [15,16]. In our patient, the pathological analysis of the lesion removed from the spinal canal confirmed the typical findings related to tophi (Figure 2).

According to Zhang T et al., most cases of intraspinal tophi are identified by neurosurgeons (19.7%), rheumatologists (19.7%), orthopedic surgeons (17.6%), and radiologists (17.6%) [3]. Therefore, these specialists should be properly trained to optimize the early diagnosis of this disorder. In the patient presented here, the presence of intraspinal tophi was suspected by the neurosurgeons, supported by the radiologists, and confirmed by the pathologists.

Once the intraspinal tophi picture is established, it is essential to assess the patient comprehensively and provide adequate treatment. If the patient has a spinal cord or root compression that produces progressive neurological deterioration, surgical decompression of the spinal canal could be necessary [3,16,20]. The surgical removal of gout tophi usually yields good results. In a systematic review of 127 studies, Zhang T et al. [3] reported that 23.9% of the patients were managed with surgery while 41.5% were treated conservatively. Clinical recovery or improvement of the neurological symptoms was achieved in 79.4% of the cases treated with surgery. In agreement with these reports, our patient responded well to the surgical intervention, improving some of the clinical symptoms.

It is important to emphasize that surgical treatment only alleviates the neurological symptoms caused by compression. The surgery should be complemented with the same clinical management as tophaceous gout of the peripheral joints [5]. Strict metabolic control of the underlying disease is required to avoid the formation of new tophi and the possible recurrence of the neurological symptoms.

## Conclusions

The patient reported in this article displayed the typical profile of neurological complications associated with axial gouty arthritis. The actual incidence of this disorder is unknown but it is probably higher than usually considered. CT studies of the spine are mandatory in patients with spinal cord involvement associated with chronic tophaceous gout. A timely diagnosis and early surgical decompression can decrease or reverse the neurological symptoms. Further studies are needed to characterize this disorder.
